# Morphological and molecular characterization of a *Sarcocystis* *bovifelis*-like sarcocyst in American beef

**DOI:** 10.1186/s13071-024-06628-4

**Published:** 2024-12-28

**Authors:** Aditya Gupta, Larissa S. de Araujo, Andrew Hemphill, Asis Khan, Benjamin M. Rosenthal, Jitender P. Dubey

**Affiliations:** 1 United States Department of Agriculture, Agricultural Research Service, Beltsville Agricultural Research Centre, Animal Parasitic Diseases Laboratory, Beltsville, MD 20705-2350 USA; 2 Institute of Parasitology, University of Bern, 3012 Bern, Switzerland

**Keywords:** *Sarcocystis*, Cattle (*Bos* spp.), Transmission electron microscopy, Molecular, Phylogenetic, *Sarcocystisbovifelis*

## Abstract

**Background:**

Parasites in the apicomplexan genus *Sarcocystis* infect cattle worldwide. Assessing the economic importance of each such parasite species requires proper diagnosis. *Sarcocystis* *cruzi,* a thin-walled species, infects virtually all cattle. The prevalence of the other thin-walled parasite, *Sarcocystis* *heydorni*, remains less well established. The remaining six species all have thick (> 3 µm) cyst walls (*Sarcocystis* *hirsuta,* *S.* *hominis,* *S.* *bovifelis,* *S.* *bovini,* *S.* *sigmoideus*, and *S.* *rommeli*). Thick-walled sarcocysts often induce inflammation in striated muscles (causing bovine eosinophilic myositis), leading to condemnation of carcasses at slaughter. One of these, *S.* *hirsuta*, can be seen macroscopically and lead to condemnation of beef. Two *Sarcocystis* species, *S.* *hominis* and *S.* *heydorni*, are zoonotic. Although *S.* *hominis* has been reported as prevalent in Europe, the occurrence of thick-walled species in the US remains poorly known. Here, for the first time to our knowldge, we characterize a thick-walled *Sarcocystis* species from a sample of beef from a local grocery store in Maryland. By morphological and genetic criteria, it closely, but not perfectly, resembles parasites previously ascribed to *S.* *bovifelis*.

**Methods:**

Beef samples were examined for *Sarcocystis* infection, using acid-pepsin digestion to search for bradyzoites, microscopically by compression between a glass slide and coverslip, by histology of paraffin embedded sections stained with hematoxylin and eosin, and by transmission electron microscopy (TEM). Molecular characterization was attempted employing genetic markers: *18S* rRNA, *28S* rRNA, *cox1*, *ITS1*, *gapdh1*, *ron3*, and *rpoB*.

**Results:**

Molecular evaluation revealed 100% identity with *S.* *bovifelis*-like sarcocysts from naturally infected cattle from Germany and Argentina; although the condition of the frozen material precludes complete characterization by TEM, we noted morphological features which differed from the *S.* *bovifelis* originally described from experimentally infected cattle from Germany.

**Conclusions:**

A novel *Sarcocystis* species is described from beef from the USA but not named until further evaluation.

**Graphical Abstract:**

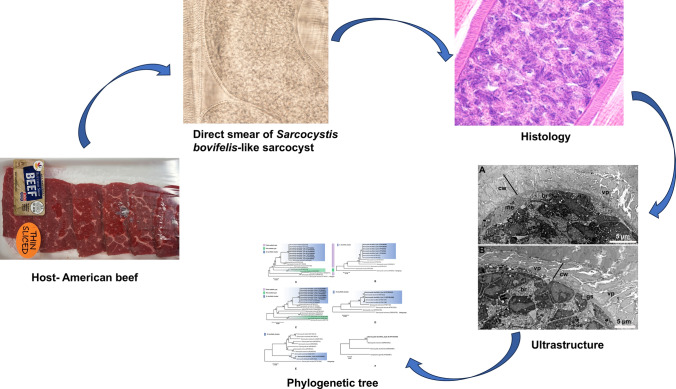

## Background

*Sarcocystis* infections in cattle are ubiquitous worldwide, attributable to an array of parasite species that are difficult to differentially diagnose. These species differ in their prevalence and pathogenicity. Proper diagnosis of *Sarcocystis* spp. is important to assess the economic and public health importance of each. Currently, there are eight named species infecting cattle: *Sarcocystis* *hirsuta,* *S.* *cruzi,* *S.* *hominis,* *S.* *bovifelis,* *S.* *heydorni,* *S.* *bovini,* *S.* *rommeli*, and *S.* *sigmoideus* [1, 2]. Canids are definitive hosts for *S.* *cruzi*, the species most pathogenic to cattle. Two species, *S.* *hominis* and *S.* *heydorni*, employ humans as their definitive host. Cats are definitive hosts for *S.* *bovifelis*, *S.* *hirsuta*, and *S.* *rommeli*. The definitive hosts for *S.* *bovini* and *S.* *sigmoideus* are unknown. Of these *Sarcocystis* species, *S.* *hirsuta* sarcocysts can become macroscopic and can lead to condemnation of beef on aesthetic grounds [3]. Additionally, *Sarcocystis* infections have been linked to an inflammatory condition of striated muscles termed bovine eosinophilic myositis (BEM). Cattle affected by BEM appear clinically normal. Diagnosis of BEM at slaughter occurs when inspecting the carcass surface or once the carcass has been divided into prime cuts or quarters. The etiology of BEM is uncertain, because the condition has not been reliably induced experimentally in cattle. Of these *Sarcocystis* species, the full endogenous life cycle stages are known only for *S.* *cruzi* [4]. The life cycle of *S.* *bovifelis* is partly known, but key studies were performed 4 -5 decades ago, before the advent of DNA sequencing necessary to confirm the identity of the etiological agent [5].

Among these eight *Sarcocystis* species, *S.* *cruzi* and *S.* *heydorni* are morphologically distinct because their sarcocysts are microscopic and have thin walls (< 1 µm thick). Sarcocysts of the remaining six *Sarcocystis* species have thick walls (> 3 µm). Differential diagnosis among those with thick walls is important given the zoonotic potential of some species and the economic costs imposed by BEM. Whereas in Europe, thick-walled and zoonotic species have been documented at considerable prevalence, little is known of the presence of thick-walled sarcocysts in the USA.

A survey for *Sarcocystis* species in American beef is in progress at the Animal Parasitic Diseases Laboratory (APDL), United States Department of Agriculture (USDA), Beltsville, Maryland. This survey involves testing beef for *Sarcocystis* bradyzoites in muscle digests, searching for sarcocysts in unstained muscle squashes, searching for sarcocysts histologically, and characterizing *Sarcocystis* species via DNA sequencing and transmission electron microscopy (TEM). As part of this study, we identified thick-walled sarcocysts in histological sections of one beef sample purchased from a grocery store. The sarcocyst wall appeared different from those previously described from beef; hence, the present detailed investigation was undertaken to characterize this thick-walled sarcocyst in the American beef. Collectively, our data identify a parasite corresponding closely, but not perfectly, to prior genetic and morphological descriptions for *S.* *bovifelis*. The available materials, once frozen, preclude comprehensive ultrastructural characterization. The molecular data provide a reference for future studies seeking to determine the prevalence and identity of this thick-walled species.

## Methods

### Sample

The beef sample was a Top Round package weighing around 500 g bought on July 23, 2023, from a local grocery store in Maryland. After trimming, a 100 g sample was tested for *Sarcocystis*. Fifty grams of muscle was ground in a meat grinder and digested in acid pepsin for 1 h; the digest was examined microscopically for *Sarcocystis* bradyzoites as previously described [6]. No sarcocysts were found in 30 muscle squashes examined microscopically, but (per our standard protocol) 50 g of muscle was stored at − 80 °C for future investigation.

### Histologic evaluation

Pieces of muscle were fixed in 10% buffered formalin and processed for histological preparation. Paraffin-embedded sections were stained with hematoxylin and eosin and examined microscopically. All sarcocysts found in 4–6 pieces of paraffin-embedded muscle (each measuring ~ 1 cm × 1 cm, enough to fill ~ 4 × 2 cm area of a histological slide) were examined at 100 × and 1000 × magnifications.

### Examination of sarcocysts from frozen, unfixed muscle samples

Frozen sample of beef was thawed at room temperature, squashed between glass slides and coverslip, and examined microscopically. Sarcocysts were photographed, numbered, and (after removing the coverslip) either saved in PBS (cyst numbers) for DNA extraction or fixed in commercial Trump’s fixative (2% paraformaldehyde, 2.5% glutaraldehyde in 100 mM phosphate buffer pH 6.8). Unfixed bradyzoites mechanically released from sarcocysts were also photographed.

### Transmission electron microscopy

Small pieces of infected beef in Trump's fixative were sent by air to the Institute of Parasitology, University of Bern, Switzerland, for TEM examination as described [2].

### DNA isolation and amplification

Genomic DNA was extracted from seven thick-walled, microscopically confirmed sarcocysts (#7, #8, #9, #10, #25, #26, #28) using the Qiagen DNeasy^®^ Blood and Tissue Kit (Hilden, Germany) following the manufacturer’s instructions. PCR amplification of highly conserved regions of *18S* *rRNA*, *28S* *rRNA*, a mitochondrial cytochrome c oxidase subunit 1 (*cox1*) and the highly variable internal transcribed spacer-1 (*ITS1*), rhoptry neck protein 3 (*ron3*), which encodes a protein that is part of rhoptry organelle, glyceraldehyde-3-phosphate dehydrogenase 1 (*gapdh1*), and a highly conserved housekeeping gene, *rpoB*, which encodes the beta-subunit of RNA polymerase, was performed using *Sarcocystis*-specific primers designed during the study (Table 1) and primers designed using isolates of *Sarcocystis* *sigmoideus* as reference (for *18S*: 310F-CGGGTAACGGGGAATTAGGG and 840R-CGTGCAGCCCAGAACATCTA; for *cox1*: 204F-TGTCGAATGTGGTGCGGTAT and 218R-AGTACCTCCCAGGCTGAACA). The 13 µl PCR mix consisted of a 2 μl DNA template, 6 μl of Platinum Hot Start PCR Master mix (Invitrogen, USA), 0.5 μl of 10 pmol/μl of each primer (IDT, USA) (Table 1), and 4 μl of molecular grade water. After initial denaturation at 94 °C for 3 min, 35 cycles were performed consisting of denaturation at 94 °C for 30 s, annealing at 60 °C for 30 s, and elongation at 68 °C for 20 min; terminal elongation incubated products at 68 C for 5 min. The PCR products were analyzed on a 2% agarose gel, and size was estimated by comparison with the 100-bp Plus DNA Ladder. The obtained PCR products were purified using the ExoSAP method [7]. The final purified PCR products were sent for sequencing to Psomagen (Rockville, MD, USA) for direct sequencing on an ABI 3500xl Genetic Analyzer (Applied Biosystems™).

**Table 1:** Accession numbers for various gene markers of *Sarcocystis* *bovifelis* infections in American beef

Gene	Cyst no.	Bases amplified	GC content (%)	Primer name	Sequence (5′–3′)	Usage	GenBank accession no.	References
*18S*	7	808	46.3	S3F	TTGTTAAAGACGAACTACTGCG	PCR amplification and sequencing	PP380089	[20] [21]
				BSarc	GATCCTTCCGCAGGTTCACCTAC	PCR amplification and sequencing		
	8	768	46.7	S3F	TTGTTAAAGACGAACTACTGCG	PCR amplification and sequencing	PP380090	[20] [21]
				BSarc	GATCCTTCCGCAGGTTCACCTAC	PCR amplification and sequencing		
	9	815	46.5	S3F	TTGTTAAAGACGAACTACTGCG	PCR amplification and sequencing	PP380092	[20] [21]
				BSarc	GATCCTTCCGCAGGTTCACCTAC	PCR amplification and sequencing		
	10	795	46.8	S3F	TTGTTAAAGACGAACTACTGCG	PCR amplification and sequencing	PP380093	[20] [21]
				BSarc	GATCCTTCCGCAGGTTCACCTAC	PCR amplification and sequencing		
	25	847	46	S3F	TTGTTAAAGACGAACTACTGCG	PCR amplification and sequencing	PP380094	[20] [22] [21]
				D23r	ATTTCTCATAAGGTGCAGGAG	PCR amplification and sequencing		
				BSarc	GATCCTTCCGCAGGTTCACCTAC	PCR amplification and sequencing		
	26	843	46.1	S3F	TTGTTAAAGACGAACTACTGCG	PCR amplification and sequencing	PP380095	[20] [21]
				BSarc	GATCCTTCCGCAGGTTCACCTAC	PCR amplification and sequencing		
	28	804	46.4	S3F	TTGTTAAAGACGAACTACTGCG	PCR amplification and sequencing	PP380097	[20] [21]
				BSarc	GATCCTTCCGCAGGTTCACCTAC	PCR amplification and sequencing		
*28S*	7	411	43.1	KL1	TACCCGCTGAACTTAAGC	PCR amplification and sequencing	PP389383	[23]
				KL3	CCACCAAGATCTGCACTAG	PCR amplification and sequencing		
	8	637	41	KL1	TACCCGCTGAACTTAAGC	PCR amplification and sequencing	PP389384	[23]
				KL3	CCACCAAGATCTGCACTAG	PCR amplification and sequencing		
	9	455	41.5	KL1	TACCCGCTGAACTTAAGC	PCR amplification and sequencing	PP389385	[23]
				KL3	CCACCAAGATCTGCACTAG	PCR amplification and sequencing		
	10	640	41.1	KL1	TACCCGCTGAACTTAAGC	PCR amplification and sequencing	PP389386	[23]
				KL3	CCACCAAGATCTGCACTAG	PCR amplification and sequencing		
	26	644	41	KL1	TACCCGCTGAACTTAAGC	PCR amplification and sequencing	PP389388	[23]
				KL3	CCACCAAGATCTGCACTAG	PCR amplification and sequencing		
*cox1*	7	862	51.3	249F	GGCAAGGATGTTTGCCATCG	PCR amplification and sequencing	PP392691	Designed during the study
				219F	TACAGCGGTATTCGTTGGGG	Sequencing		
				198R	CAGGCTGAACAGCAGTACGA	PCR amplification and sequencing		
	8	876	51	249F	GGCAAGGATGTTTGCCATCG	PCR amplification and sequencing	PP392692	Designed during the study
				219F	TACAGCGGTATTCGTTGGGG	Sequencing		
				198R	CAGGCTGAACAGCAGTACGA	PCR amplification and sequencing		
	9	901	51.1	249F	GGCAAGGATGTTTGCCATCG	PCR amplification and sequencing	PP392693	Designed during the study
				219F	TACAGCGGTATTCGTTGGGG	Sequencing		
				198R	CAGGCTGAACAGCAGTACGA	PCR amplification and sequencing		
	10	886	51.1	249F	GGCAAGGATGTTTGCCATCG	PCR amplification and sequencing	PP392694	Designed during the study
				219F	TACAGCGGTATTCGTTGGGG	Sequencing		
				198R	CAGGCTGAACAGCAGTACGA	PCR amplification and sequencing		
	25	893	51.2	249F	GGCAAGGATGTTTGCCATCG	PCR amplification and sequencing	PP392695	Designed during the study
				219F	TACAGCGGTATTCGTTGGGG	Sequencing		
				198R	CAGGCTGAACAGCAGTACGA	PCR amplification and sequencing		
	26	863	51.2	249F	GGCAAGGATGTTTGCCATCG	PCR amplification and sequencing	PP392696	Designed during the study
				219F	TACAGCGGTATTCGTTGGGG	Sequencing		
				198R	CAGGCTGAACAGCAGTACGA	PCR amplification and sequencing		
	28	866	50.9	249F	GGCAAGGATGTTTGCCATCG	PCR amplification and sequencing	PP392697	Designed during the study
				219F	TACAGCGGTATTCGTTGGGG	Sequencing		
				198R	CAGGCTGAACAGCAGTACGA	PCR amplification and sequencing		
*ITS1*	8	157	43.9	150F	CACACCTTCTTTCCCTCTGCT	PCR amplification and sequencing	PP391620	Designed during the study
				162R	TAGCAGCATTCCTGTCTCCAG	PCR amplification and sequencing		
	10	171	43.3	150F	CACACCTTCTTTCCCTCTGCT	PCR amplification and sequencing	PP391621	Designed during the study
				162R	TAGCAGCATTCCTGTCTCCAG	PCR amplification and sequencing		
*gapdh1*	25	846	52.4	SeqGAPDH1F	AACGGTGCGAAGAAGGTGAT	PCR amplification and sequencing	PP392698	Designed during the study
				SeqGAPDH1R	TACCAGCACGCCAGTCTTTT	PCR amplification and sequencing		
*ron3*	25	505	49.3	SeqRON3F	CAAAACGGTTTGCGGGGAAT	PCR amplification and sequencing	PP392699	Designed during the study
				SeqRON3R	CGCTTGGCTCGGAAAATACG	PCR amplification and sequencing		
*rpoB*	25	775	22.3	rpoBF	AACAGTAGGTGACAAATTATGTGGT	PCR amplification and sequencing	PP748570	Designed during the study
				rpoBR	AGAATACCTTGCAACTCCACAA	PCR amplification and sequencing		

Trimmed and annotated sequences belonging to *18S,* *28S,* *cox1,* *ITS1,* *gapdh1,* *ron3*, and *rpoB* were aligned using the Clustal W 2.1 alignment tool [8] implemented within Geneious Prime 2023.0.4 (www.geneious.com) and were submitted to GenBank (Table 1). Species identity was verified using the Basic Local Alignment Search Tool (BLAST) [9]. The final edited sequence length of each gene marker and their GC content appear in Table 1.

The web server Guidance 2 [10] was used to align and remove ambiguously aligned positions in each gene. The sequences were aligned with the MAFFT algorithm under the options Max-Iterate: 1000 and Pairwise Alignment Method: -localpair. Positions with a score < 0.93 were removed as well as positions with > 25% missing data. A model test was performed using jModelTest 2.1.7 [11] to determine the most suitable nucleotide substitution model, according to the Bayesian information criterion (BIC). Phylogenetic relationships were reconstructed under the maximum likelihood (ML) criteria. ML analyses were performed with the program IQ-TREE version 1.6.12 [12]. The analyses were run with the options -m MFP -b 1000 -nt 5 (i.e., ModelFinder + tree reconstruction + 1000 non-parametric bootstrap replicates + tree topology test). The model selected based on the BIC criterion was (*18S* = K2 + G, lowest BIC = 3405.9; *28S* = K2 + G, lowest BIC = 2256.7; *cox1* = K2 + G, lowest BIC = 9803.7; *ITS1* = HKY, lowest BIC = 2815.4, *rpoB* = T92 + G, lowest BIC = 3446.1 and *gapdh1* = T92 + I, lowest BIC = 4849.7). The nucleotide frequencies were also estimated for each gene.

## Results

### Light microscopy

In the original histological slide, six thick-walled sarcocysts were detected in two of six muscle pieces. Thereafter, an intensive search was made to find thick-walled sarcocysts. An additional 80 pieces of formalin-fixed samples of muscle, embedded in 16 paraffin blocks, were processed. From these, a total of two sarcocysts were detected in 1 of the 16 histological slides. These 17 paraffin blocks were cut a second time, yielding only 8 cysts. A third recut of 17 blocks revealed only 1 cyst. Thus, a total of 17 thick-walled sarcocysts were detected in 51 histological sections. One sarcocyst was thin-walled and appeared to be *S.* *cruzi* (excluded from further study).

**Fig. 1:**
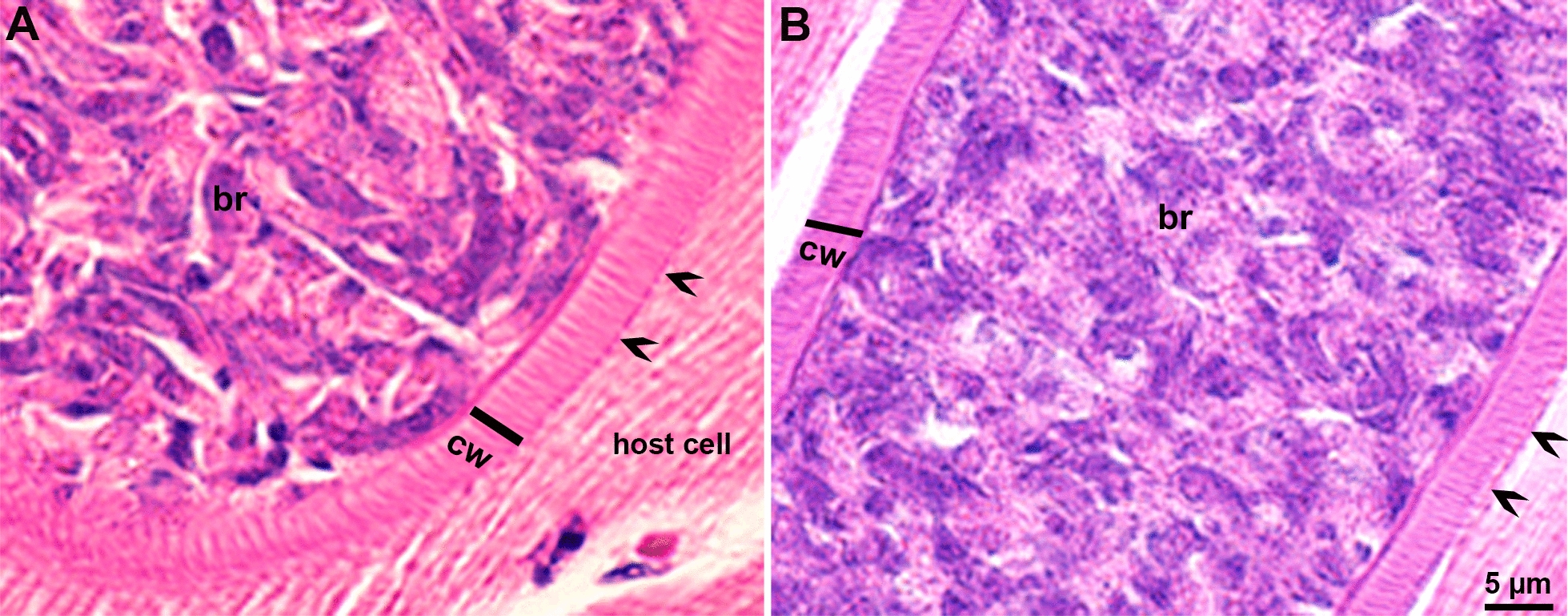
**A, B** Two mature sarcocysts in histological sections of beef. Note thick cyst walls (cw), straight to slopping villar protrusions (vp) densely staining villar protrusion ends (arrowheads). Hematoxylin-eosin stain

The thick-walled sarcocysts in H&E-stained histological sections were 38–400 × 26–75 µm. The sarcocyst wall was 2.8–5.6 µm thick and contained villar protrusions that were upright to sloping; their tips were thickened (Fig. 1).

### Examination of sarcocysts from frozen, unfixed samples

**Fig. 2:**
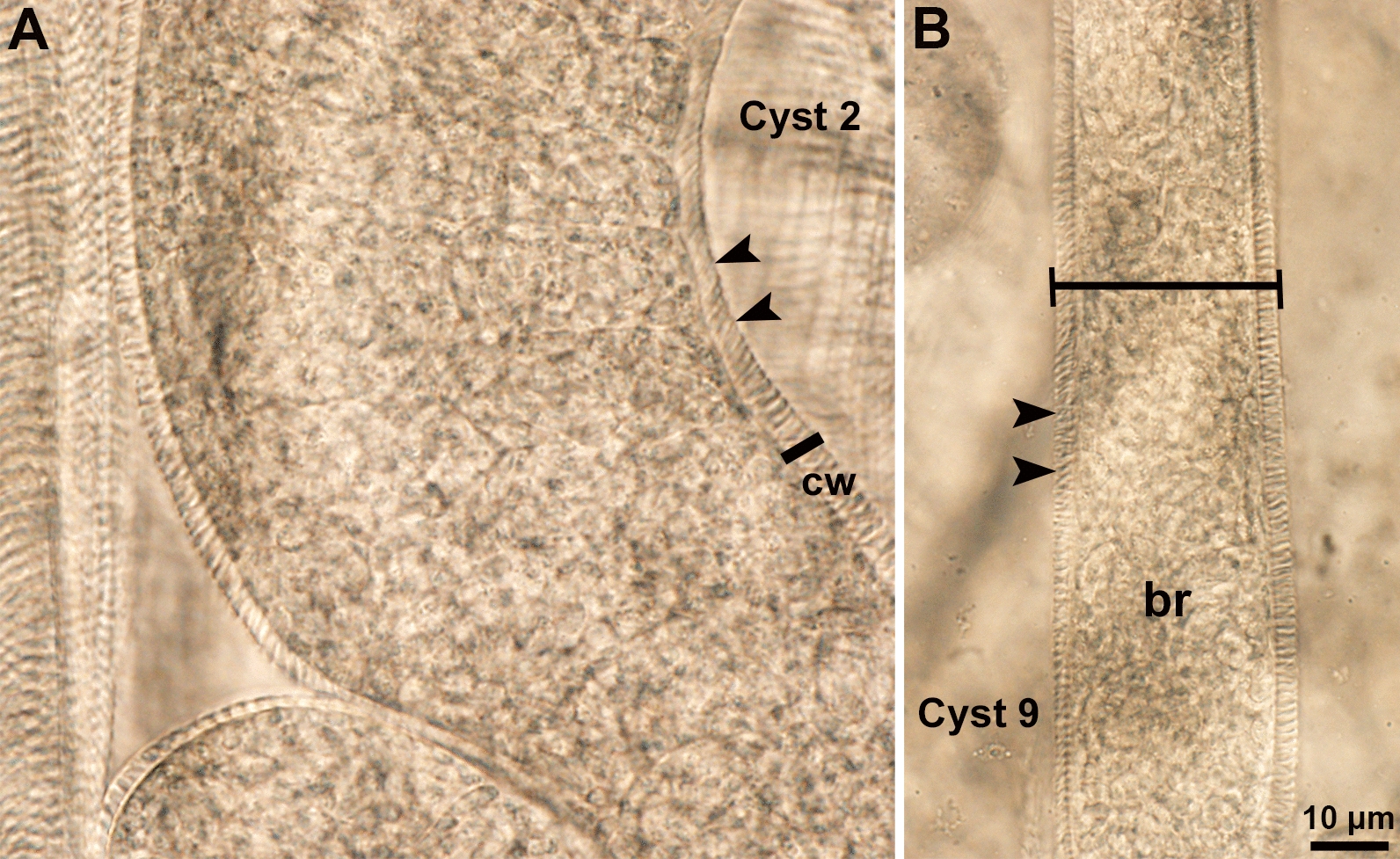
**A, B** Two mature sarcocysts in muscle compressed between a glass slide and cover slip. Note variability of the thickness of cysts (cyst marked 9 is slender compared with cyst marked 2) and increased density of tips of villar protrusions (arrowheads). Unstained

From several grams of frozen muscle, a total of 44 sarcocysts were found (3 of which appeared thin-walled; resembling *S.* *cruzi,* they were not studied further). All the remaining 41 thick-walled sarcocysts were < 1 mm long, except one that was 1860 µm long and 43 µm wide. The sarcocyst wall was 3.4–6.7 µm, depending upon the flattening of the cyst and angle measured (Fig. 2). Free bradyzoites were 12–14 × 3–4 µm.

### TEM

**Fig. 3:**
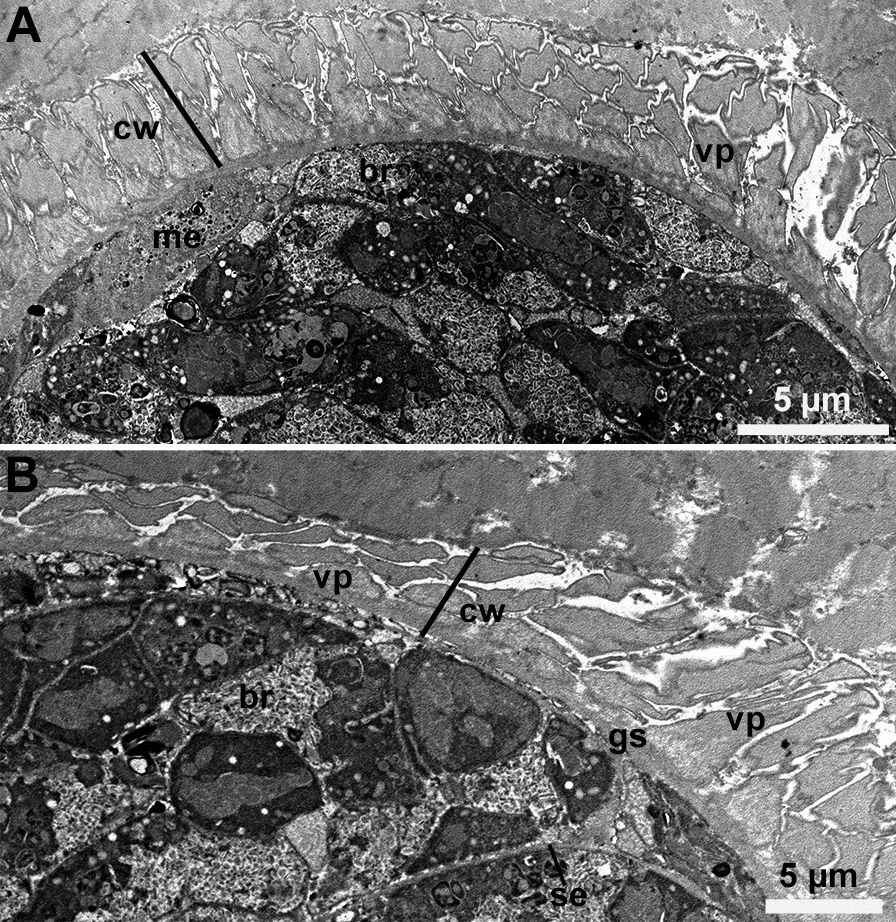
Transmission electron micrographs of sarcocyst #38. **A, B** Note variability of the appearance of villar protrusions (vp) and the thickness of the cyst wall (cw). The ground substance layer (gs) is thin and continues into the interior of the sarcocyst as septa (se). Bradyzoites (br) and metrocytes (me) are juxtaposed with the gs

**Fig. 4:**
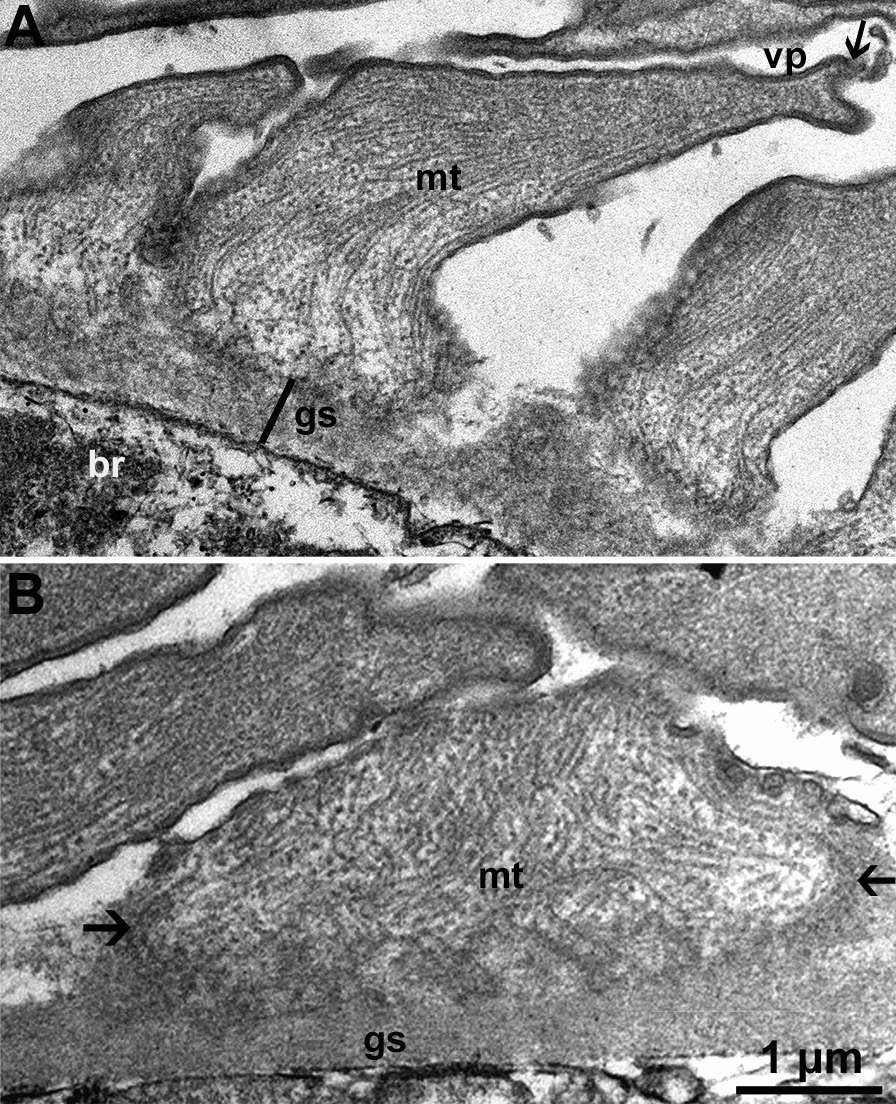
Transmission electron micrographs of two sarcocysts. **A** Longitudinal section of a villar protrusion (vp). Note the ground substance layer (gs) is thin and devoid of granules. The vp has a broad base and forked, tapered tip (arrow). The microtubules (mt) in vp arise at the base of gs; they are devoid of granules and extend up to half of the length of the vp. Note part of degenerated bradyzoite (br) at the base of gs. Cyst #34. **B** Higher magnification of the base of villar protrusion to show absence of vesicles and the origin of microtubules (mt). Cyst #38

**Fig. 5:**
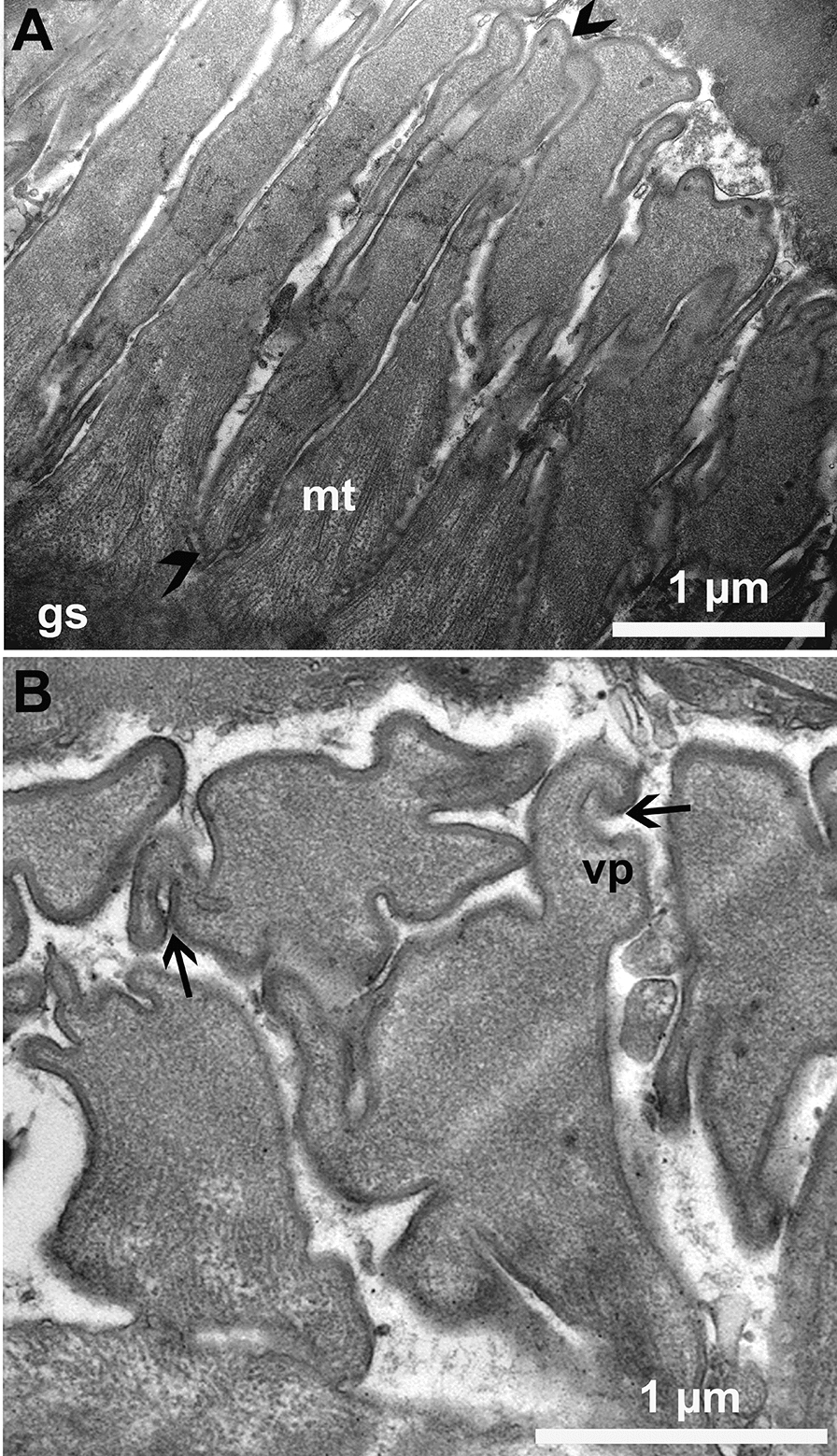
Transmission electron micrographs of two sarcocysts. **A** Longitudinal section of a villar protrusion (vp) (opposing arrowheads). Cyst #38. **B** Sections through villar tips (vp) to show hooks at the tips (arrows). Note absence of microtubules at the villar tips. Cyst #13

Six sarcocysts were studied by TEM. Although sarcocysts were sub-optimally preserved because the specimens had been frozen, the sarcocyst wall structures were preserved. The thickness of the sarcocyst wall varied with the plane of the section (Fig. 3). The ground substance was indistinct, < 0.5 µm thick, and devoid of granules or tubules (Fig. 4). The villar protrusions (vp) were elongated, mostly sloping, up to 5.8 µm long (Fig. 5). The vp were broad at the base, up to 1.8 µm in tangentially cut section, and tapered distally. The vp tips were often forked or had hook-like structures (Figs. 4, 5). The vp were lined with fine microtubules that criss-crossed at the base and extended no more than one-half length of the vp (Figs. 4, 5).

### Molecular analysis

The selected genetic markers (*18S,* *28S,* *cox1,* *ITS1,* *gapdh1*, *ron3*, and *rpoB*) were successfully amplified and sequenced. The obtained sequences under study showed nearly 100% identity to *S.* *bovifelis* described from Argentina, Lithuania, and Kazakhstan. No published reference exists for *rpoB* in *S.* *bovifelis;* our sequence showed < 99% identity to that reported for *Sarcocystis* *falcatula* (Table 2). Moreover, the primers intended to specifically amplify *S.* *sigmoideus* amplified a product from these seven cysts, but the resulting sequence matched that expected for *S.* *bovifelis*.

**Table 2:** Homogeneity of various gene marker sequences of thick-walled *Sarcocystis* spp. infecting muscles of cattle available in the GenBank with *Sarcocystis* *bovifelis* isolated during the study

Species	Gene	Accession no.	Country	Query cover (%)	DNA sequence identity to *S.* *bovifelis*	Gaps	References
*Sarcocystis* *bovifelis*	*18S*	KT901136	Argentina	100	865/865 (100%)	0	[15]
		KT901137	Argentina	100	865/865 (100%)	0	
	*28S*	KT901247	Argentina	100	642/644 (99.69%)	0	
		KT901255	Argentina	100	641/644 (99.53%)	0	
	*cox1*	KC209695	Argentina	100	934/935 (99.89%)	0	
		KT900992	Argentina	100	934/935 (99.89%)	0	
	ITS1	KT901197	Argentina	100	170/171 (99.42%)	0	
		KT901198	Argentina	100	170/171 (99%)	0	
*Sarcocystis* *bovini*	*18S*	KT901149	Argentina	100	861/865 (99.54%)	0	
	*28S*	KT901257	Argentina	99	628/639 (98.28%)	0	
	*cox1*	KT901017	New Zealand	100	877/935 (93.80%)	0	
	ITS1	KT901203	Argentina	100	171/171 (100%)	0	
*Sarcocystis* *rommeli*	*18S*	KY120284	China	100	861/865 (99.54%)	0	[24]
	*28S*	ON229491	China	95	587/614 (95.60%)	4	-
	*cox1*	KY120292	China	97	853/907 (94.05%)	0	[24]
*Sarcocystis* *hirsuta*	*18S*	KT901166	Germany	100	853/877 (97.26%)	15	[15]
	*28S*	KT901269	New Zealand	100	590/654 (90.21%)	20	
	*cox1*	KT901024	China	97	853/907 (94.05%)	0	
	ITS1	KT901220	Brazil	14	20/24 (83.33%)	0	
*Sarcocystis* *hominis*	*18S*	KF954731	Germany	100	857/867 (98.85%)	2	[25]
	*cox1*	OR543021	Italy	94	778/890 (87.42%)	14	[2]
*Sarcocystis* *sigmoideus*	*18S*	OR526731	Italy	95	811/844 (96.09%)	18	[2]
		OR526732	Italy	100	848/881 (96.25%)	20	
	*cox1*	OR543013	Italy	93	706/877 (80.50%)	2	
		OR543014	Italy	93	708/877 (80.93%)	2	

### Phylogenetic analysis

**Fig. 6:**
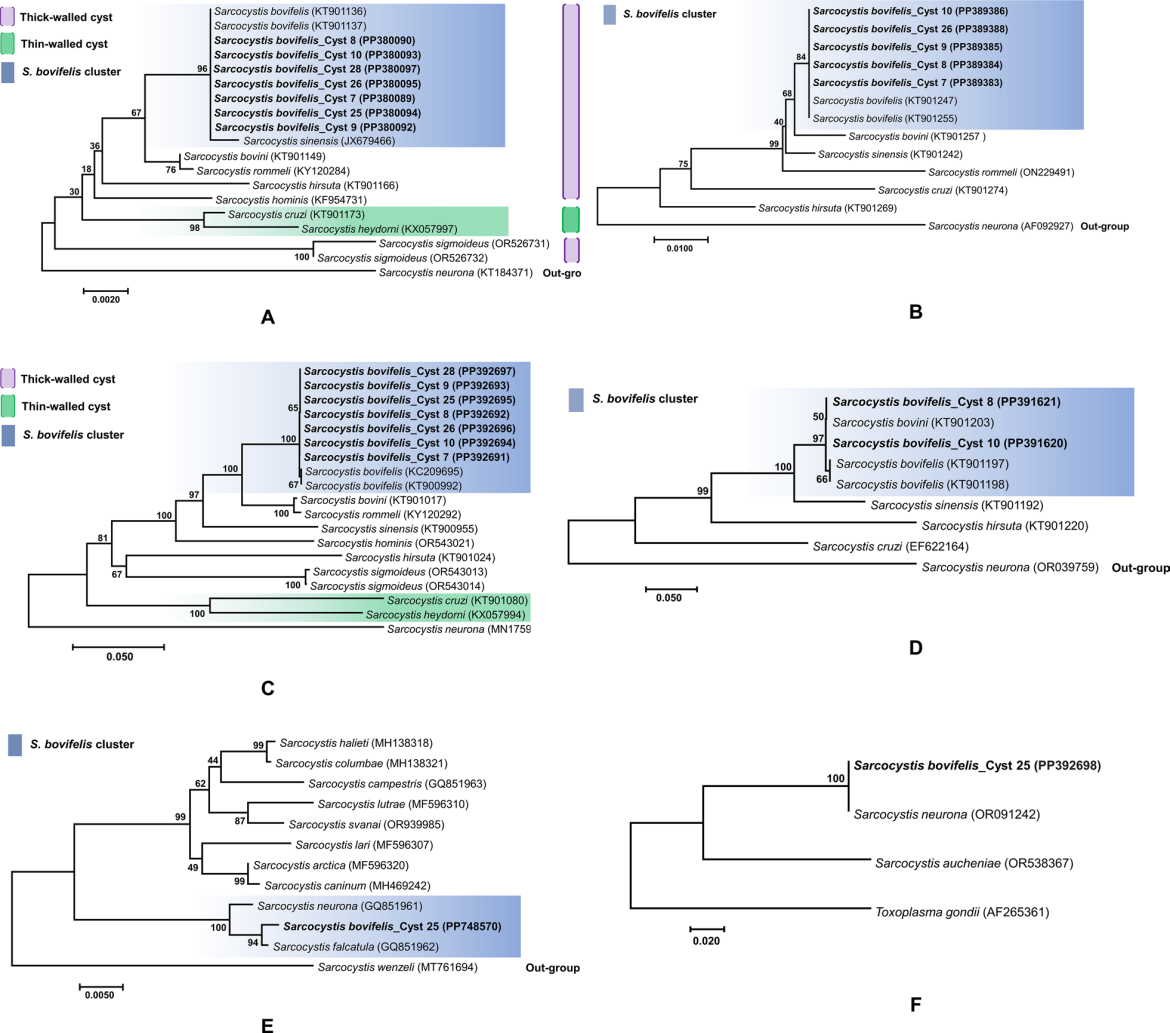
Phylogenetic relationships of *Sarcocystis* *bovifelis* with various *Sarcocystis* species infecting cattle inferred from different genetic markers (**A** = *18S* *rRNA*, **B** = *28S* *rRNA*, **C** = *cox1*, **D** = *ITS1,* **E** = *rpoB* and **F** = *gapdh1*) under maximum likelihood criterion (ModelFinder + tree reconstruction + 1000 non-parametric bootstrap replicates + tree topology test). Branch supports are indicated near the corresponding nodes. The highlighted part (blue) shows the *S.* *bovifelis* cluster; pink shows the thick-walled cyst cluster, and green part shows the thin-walled cyst cluster with highest nodal support. Species in bold are the ones obtained during the study

The final phylogenetic analyses included 19 taxa and 1856 positions for *18S*, 13 taxa and 417 positions for *28S*, 19 taxa and 1041 positions for *cox1*, 9 taxa and 1066 positions for *ITS1*, 12 taxa and 783 positions for *rpoB*, and 4 taxa and 846 positions for *gapdh1* from the lineage of Sarcocystidae and different isolates of *Sarcocystis* *neurona*, *Toxoplasma* *gondii* (for *gapdh1* gene) and *Sarcocystis* *wenzeli* (for *rpoB* gene) as outgroups. The phylogenetic trees for the six loci were reconstructed and analyzed based on maximum likelihood and Bayesian criteria for the positioning of the species under study in the lineage of *Sarcocystis* species infecting cattle, primarily the thick-walled sarcocysts. The trees reconstructed only differ in the position of a few low-supported branches showing close homology between the *Sarcocystis* species infecting cattle (Fig. 6). The tree based on all the genetic markers utilized during the study included all available data from species infecting cattle (i.e. *S.* *bovini*, *S.* *bovifelis*, *S.* *cruzi*, *S.* *heydorni*, *S.* *hirsuta*, *S.* *hominis*, *S.* *rommeli*, and *S.* *sigmoideus*).

Apparently, the phylogenetic tree for each genetic marker distinguished, with strong bootstrap support, thick- from thin-walled species of *Sarcocystis* infecting cattle (Fig. 6), consistent with previous findings [13–15].

For the *18S* and *cox1* genes, there were two clusters, one thick- and the other thin-walled with high consensus support showing the tendency of tissue specificity for *Sarcocystis* species. The sequences isolated from all seven cysts clustered with different isolates of *S.* *bovifelis* described from Argentina based on the best match (which has been called as “*S.* *bovifelis* cluster” in the tree) in a single clade with high bootstrap values. This cluster also includes other thick-walled *Sarcocystis* species including *S.* *bovini*, *S.* *rommeli*, *S.* *hirsuta*, and *S.* *hominis*, the other cluster being thin-walled consisting of *S.* *cruzi* and *S.* *heydorni*.

For other genes (*28S*, *ITS1* and *rpoB*), due to less data availability of *S.* *cruzi* and *S.* *heydorni* at the NCBI, only thick-walled cluster and the “*S.* *bovifelis* cluster” could be studied.

## Discussion

Based on molecular characterization, the thick-walled *Sarcocystis* species in the present study most closely resembled *S.* *bovifelis*, but with some differences (SNP’s or single nucleotide polymorphism). Some of this confusion and uncertainty may never be resolved because of inadequate original description of *S.* *bovifelis*. Therefore, it is important to document the basis for the original description of *S.* *bovifelis*.

In 1975, Heydorn et al. [16] proposed a new nomenclature for naming *Sarcocystis* species in livestock. When naming *S.* *bovifelis* [16], all cattle *Sarcocystis* species transmitted by cats were then attributed to one species, *S.* *bovifelis*. Its description was based on characterizing sarcocysts in experimentally infected cattle [17]. For this, six weaned calves were orally inoculated with sporocysts from the feces of eight cats that had been fed the esophagi of naturally infected cattle in Germany (details were translated into English [5]). Transmission electron microscopic observations were based on two calves necropsied 98 and 60 days post inoculation (p.i.). Relevant details are restated here. The villar protrusions (vp) on the sarcocyst wall were 3.8–5.4 long; the original description makes no mention of thickening of the villar tips [17]. The vp were sloping, broader at the base, and tapered distally, but the dimensions were not specified. Microtubules were present throughout the vp. Additionally, vesicles were stated to be present at the base of the vp [17]. However, of the five illustrations of vp [17], four show sloping vp but no vesicles at the base. One image (Fig. 8 of [17]) shows a sarcocyst with vp that have straight protrusions with flattened tips and the presence of vesicles at the base of vp. Better quality illustrations of the cyst wall of *S.* *bovifelis* from the same experiment were provided by Mehlhorn et al. [18]. The vp were broader at the base and tapered distally. In all three illustrations, no vesicles are evident at the base of vp [18]. No archived specimens remain from the original study.

**Fig. 7:**
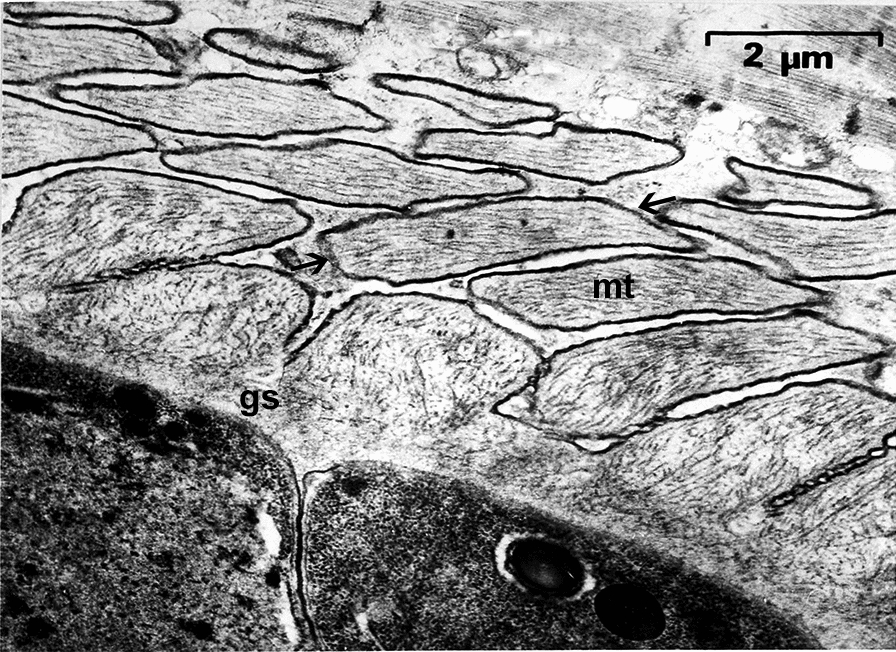
Transmission electron micrograph of a sarcocyst in muscle of a calf fed sporocysts from the feces of naturally infected cats in Germany from Gestrich et al. [17]. Note microtubules extend from the base to end of villar protrusions and the absence of any vesicles at the base of villar protrusions

The authors of the initial description [16] submitted specimens of sarcocysts of *S.* *bovifelis* and other species to several individuals and institutions, including Beltsville, Maryland, USA. Re-examination of the calf necropsied at 98 days p.i. revealed a 5.5-µm-thick sarcocyst wall but no thickening of the villar tips (Fig. 5C, D of [5]). An unpublished electron micrograph from the German study [17] provided to us is produced here (Fig. 7). It shows sloping vp but no vesicles at the vp base. It is likely that more than one *Sarcocystis* species was present in calves experimentally infected by Gestrich et al. [17]; the sporocysts were derived from feces of several cats that were fed naturally infected beef and thus could have been derived from more than one *Sarcocystis* species.

The only other study of the life cycle of a cat-transmitted *Sarcocystis* is that of Dubey [5, 19]. These parasites were isolated from muscle of an experimental cow in Bozeman, Montana, USA, and subsequently serially passed in cats and calves [19]. Newborn calves were inoculated with sporocysts from experimentally infected cats, and calves were killed at intervals to study parasite development through ensuing life cycle stages, documenting development of schizonts and sarcocysts. Only a few mature sarcocysts were present in experimentally infected calves. By light microscopy, the sarcocyst wall resembled that of [17] and villar tips were not thickened; ultrastructure of sarcocysts was not studied [5]. There are no archived paraffin blocks for molecular studies.

Gjerde [15] molecularly characterized 45 *S.* *bovifelis*-like sarcocysts from naturally infected beef from Argentina and Germany using six genes. These sarcocysts were 1–3 mm long, much longer than those reported from experimentally infected cattle; this report lacked accompanying TEM description. Gjerde [15] also named another species, *S.* *bovini*, whose sarcocysts morphologically resembled *S.* *bovifelis* by light microscopy but which differed molecularly; again, no TEM accompanied that description. These findings are relevant for the discussion of the *Sarcocystis* species in the present study.

Features of sarcocysts of different *Sarcocystis* species by TEM are summarized in Table 3. *Sarcocystis* *hirsuta* and *S.* *hominis* sarcocysts are morphologically distinct. The ground substance layer in sarcocysts of *S.* *hirsuta* and *S.* *hominis* is thick, and their vp are also distinctive. The vp of *S.* *hirsuta* have a small stalk at the base and vp are expanded laterally. The vp of *S.* *hominis* are cylindrical. *Sarcocystis* *rommeli* sarcocysts have prominent vesicles at the base of vp; these are absent in other species (Table 3). The microtubules in vp in the species in the present study are absent in the 1/3 distal portions of vp, and there are no vesicles at the base of vp. Naming of the new species here is deferred until optimally fixed specimens are available for ultrastructural description.

**Table 3:** Comparison of cyst wall structures of thick-walled sarcocysts of cattle by transmission electron microscopy

Characteristics	*Sarcocystis* *hominis*	*Sarcocystis* *bovifelis*	*Sarcocystis* *rommeli*	*Sarcocystis* *hirsuta*	*Sarcocystis* *sigmoideus*	Present study
No. of sarcocysts studied	Many	?	4	*3*	*2*	6
**Cyst wall type**	10b	10 g	10 g	28	New type	10 g?
**Villar protrusions (vp)**						
Length of the cysts	5.0–7.5 µm long	?	5 × 0.5 µm	Up to 8 µm long	?	Up to 5.3 µm
Shape of the vp	Straight, finger-like, cylindrical tips	Broad at base, distally tapered	Broad at base, distally tapered	Narrow at base	Narrow at base	Broad at base, distally tapered
Microtubules	Whole length of the vp	Whole length of the vp	Whole length of the vp	Whole length of the vp	Whole length of the vp, granules on microtubules	One-half of vp, smooth
Vesicles at base	Absent	?	Present	Absent	Absent	Absent
**Ground substance layer thickness**	Thick (2 µm)	Thin	Thin (< 0.8 µm)	Thick (2.2 µm)	Thin (< 1 µm)	Thin (< 0.3 µm)
Main reference	[6, 26]	[17, 18]	[6]	[5]	[2]	

Thus, we document the occurrence of a thick-walled species of parasite in American beef that most closely resembles genetic signatures previously reported for *S.* *bovifelis* but which differ, ultrastructurally, from the material initially employed in the description of *S.* *bovifelis.* The available materials, once frozen, preclude definitive ultrastructural characterization. The genetic data, however, may prove useful in discerning the prevalence and identity of the thick-walled parasite herein identified.

## Data Availability

No datasets were generated or analyzed during the current study.

## Abbreviations

APDL: Animal Parasitic Diseases Laboratory
BEM: Bovine eosinophilic myositis
BIC: Bayesian information criterion
BLAST: Basic Local Alignment Search Tool
*cox1*: Cytochrome *c* oxidase subunit 1
*18S* rRNA: 18S ribosomal RNA
*28S* rRNA: 28S ribosomal RNA
*gapdh1*: Glyceraldehyde-3-phosphate dehydrogenase 1
gs: Ground substance
H&E: Hematoxylin and eosin
*ITS1*: Internal transcribed spacer-1
MAFFT: Multiple Alignment using Fast Fourier Transform
ML: Maximum likelihood
*ron3*: Rhoptry neck protein 3
*rpoB*: RNA polymerase β subunit
SNP: Single nucleotide polymorphism
TEM: Transmission electron microscopy
USDA: United States Department of Agriculture
vp: Villar protrusions
