# Correction: Morphological and molecular characterization of a *Sarcocystis bovifelis*-like sarcocyst in American beef

**DOI:** 10.1186/s13071-025-06665-7

**Published:** 2025-01-16

**Authors:** Aditya Gupta, Larissa S. de Araujo, Andrew Hemphill, Asis Khan, Benjamin M. Rosenthal, Jitender P. Dubey

**Affiliations:** 1 United States Department of Agriculture, Agricultural Research Service, Beltsville Agricultural Research Centre, Animal Parasitic Diseases Laboratory, Beltsville, MD 20705-2350 USA; 2 Institute of Parasitology, University of Bern, 3012 Bern, Switzerland

**Correction: Parasites & Vectors (2024) 17:543** 10.1186/s13071-024-06628-4

Following publication of the original article [1], it came to the journal's attention that Fig. 5 was incomplete: the panel label ‘A’ (referred to in the caption) was missing from the image of the figure. The figure has since been corrected. The publisher thanks you for reading this erratum and apologizes for any inconvenience caused.
